# The Means of Contagion in Scarlet Fever

**Published:** 1886-10

**Authors:** J. M. Keating


### *THE MEANS OF CONTAGION IN SCARLET FEVER.*

Scarlet fever can be communicated by infected milk, and, as far as we know, the milk has only to stand in the room where the disease exists or has existed to absorb the germs, which are so subtle, so light, and yet so tenacious as to float in the air and adhere to particles of dust.

We all know how much dust is constantly floating in the air; let a beam of sunlight pass through an opening in the shutter, and we can readily see how the scales of skin from the body, pieces of lint, etc., can carry these microbes, which may be thrown off in the mucus from the nostrils and mouth, or in the perspiration, and even the urine.

Not only are these secretions germ-carriers—that is contagious—and they have been all proven so by direct inoculation, but the passages from the bowels, as well as the urine, are so—in that way sewer-air may be a means of their conveyance; drinking water also, as well as the vapor from soil on which these matters have been thrown. Bear in mind, then, that the scarlatina poison can be carried in this way hundreds of miles; that it does not need the personal contact of individuals; that it retains its vitality for months' and even years, unless it be subjected to certain influences that either entirely destroy it or deprive it of its malignancy—these are intense heat, especially boiling or steam, plenty of fresh air, and certain chemical substances, as chlorine, sulphurous acid, and others. There is one other point which is important. It is now known that animals, such as horses aud dogs, have a disease which is evidently scarlatina; they can be infected by the scarlatina of man, and probably their disease can be communicated to man.

The poison of scarlatina is, then, either *inhaled* by the individual or is *swallowed*. It is then taken up by the circulation, and, finding itself surroundd by material which develops it, vivifies it, becomes rapidly reproduced, and the symptoms of the disease show themselves. This period between the reception of the poison and the appearance of the symptoms is called the period of *incubation;* this is known to be either from *one* to *six* days, in some cases longer.—*Dr. J. M. Keating, in Babyhood.*

